# Comprehensive analysis of competitive endogenous RNA associated with immune infiltration in lung adenocarcinoma

**DOI:** 10.1038/s41598-021-90755-w

**Published:** 2021-05-26

**Authors:** Wenjie Chen, Wen Li, Zhenkun Liu, Guangzhi Ma, Yunfu Deng, Xiaogang Li, Zhu Wang, Qinghua zhou

**Affiliations:** 1 Lung Cancer Center, West China Hospital, Sichuan University, Chengdu, China; 2 Department of Thoracic Surgery, West China Hospital, Sichuan University, Chengdu, China; 3 Laboratory of Molecular Diagnosis of Cancer, Cancer Center, West China Hospital, Sichuan University, Chengdu, China; 4 Tianjin Key Laboratory of Lung Cancer Metastasis and Tumor Microenvironment, Tianjin Lung Cancer Institute, Tianjin Medical University General Hospital, Tianjin, China

**Keywords:** Cancer, Tumour biomarkers

## Abstract

To identify the prognostic biomarker of the competitive endogenous RNA (ceRNA) and explore the tumor infiltrating immune cells (TIICs) which might be the potential prognostic factors in lung adenocarcinoma. In addition, we also try to explain the crosstalk between the ceRNA and TIICs to explore the molecular mechanisms involved in lung adenocarcinoma. The transcriptome data of lung adenocarcinoma were obtained from The Cancer Genome Atlas (TCGA) database, and the hypergeometric correlation of the differently expressed miRNA-lncRNA and miRNA-mRNA were analyzed based on the starBase. In addition, the Kaplan–Meier survival and Cox regression model analysis were used to identify the prognostic ceRNA network and TIICs. Correlation analysis was performed to analysis the correlation between the ceRNA network and TIICs. In the differently expressed RNAs between tumor and normal tissue, a total of 190 miRNAs, 224 lncRNAs and 3024 mRNAs were detected, and the constructed ceRNA network contained 5 lncRNAs, 92 mRNAs and 10 miRNAs. Then, six prognostic RNAs (*FKBP3, GPI, LOXL2, IL22RA1, GPR37*, and *has-miR-148a-3p*) were viewed as the key members for constructing the prognostic prediction model in the ceRNA network, and three kinds of TIICs (Monocytes, Macrophages M1, activated mast cells) were identified to be significantly related with the prognosis in lung adenocarcinoma. Correlation analysis suggested that the *FKBP3* was associated with Monocytes and Macrophages M1, and the *GPI* was obviously related with Monocytes and Macrophages M1. Besides, the *LOXL2* was associated with Monocytes and Activated mast cells, and the *IL22RA1* was significantly associated with Monocytes and Macrophages M1, while the *GPR37* and Macrophages M1 was closely related. The constructed ceRNA network and identified Monocytes, Macrophages M1 and activated Mast cells are all prognostic factors for lung adenocarcinoma. Moreover, the crosstalk between the ceRNA network and TIICs might be a potential molecular mechanism involved.

## Introduction

Lung cancer is one of the most frequently diagnosed malignancies worldwide, and it represents almost one-quarter of all cancer deaths^1^. Lung cancer is categorised into adenocarcinoma (LUAD), squamous cell type and large cell type, and LUAD accounts for more than 40% of all lung cancer cases^2^. For lung cancer patients, it is often difficult to diagnosed until an advanced disease stage, hence, the 5-year overall survival rate is still frustrating for all stages^3^. Besides, the conventional treatments, including surgery, radiotherapy, and chemotherapy, for metastatic lung cancer is remains modest, so it is vital to make a comprehensive understanding of the tumorigenesis and tumor progression and find out potential biomarkers to accurately predict the prognosis^4,5^.

Over the years, it has been generally recognized that non-coding RNA (ncRNA) have transformed the tumorigenesis and progression landscape in serious tumors. Competitive endogenous RNA (ceRNA) network, composed of mRNAs, long non-coding RNAs (lncRNAs) and microRNAs (miRNAs), is a novel class of ncRNAs and plays an important role in the post-transcriptional regulation by modulate the oncogenes or tumor suppressor genes and the interactions between protein and genes^6–8^. Moreover, studies have recently demonstrated that the ceRNA regulatory networks is a prognostic indicator in different diseases, such as cardiovascular diseases, diabetic cataract, myocardial infarction, and some malignancies, including myeloid leukemia, bladder cancer and pancreatic adenocarcinoma^9,10^.

Recently, studies have identified that the tumor immune microenvironment (TIME) plays a vital role in tumorigenesis, metastasis and could also critically influence therapeutic response. Tumor infiltrating immune cells (TIICs) which are regulated by different cytokines and chemokines consist the main regulators of tumor biology in the tumor microenvironment (TME)^11,12^. Previous study shows that TIICs, especially the tumor‐associated macrophage, are associated with oncological outcomes and drug response to Bacillus Calmette-Guerin (BCG) treatment in non‐muscle‐invasive bladder cancer^13^. Besides, it has also been shown that the TIICs could have effect on the ceRNA network by regulating the expression of non-coding RNA directly or indirectly^14^.

In the current study, we detected the different expression of ceRNA in TCGA lung adenocarcinoma and calculated the TIICs by CIBERSORT algorithm. Then, we built nomograms based on the prognosis model, which can be used to assess prognosis for lung adenocarcinoma patients. Moreover, the correlation analysis between the prognostic members in the ceRNA and prognostic TIICs was performed, our results might render to clarify the mechanism of the occurrence and development of lung adenocarcinoma combined with tumor immune microenvironment regulation.

## Methods

### Data collection and identification of differently expressed RNAs

We downloaded all the miRNA, lncRNA and mRNA expression profiles and relevant clinical data such as pathological factors, and the survival outcome of the lung adenocarcinoma cohort from The Cancer Genome Atlas (TCGA) (https://portal.gdc.cancer.gov/), including 497 cancer cases, 54 cases of adjacent normal tissues and 486 cases of relevant clinical data. Another independent validation sets GSE14814^15^ with 71 LUAD samples and common clinicopathological characteristics were obtained from the Gene Expression Omnibus (GEO) database (https://www.ncbi.nlm.nih.gov/geo/query/acc.cgi?acc=GSE14814).

The differently expressed RNAs were screened by using the R package ‘edgeR^16^’, | Log (fold change) |≥ 1.0 and *p*-value < 0.05 were set as the cut-off. To visualize the differently expressed RNAs, we used the R package ‘ggplot2’.

### Construction of the ceRNA network and functional enrichment analysis

The hypergeometric correlation of the differently expressed miRNA-lncRNA and miRNA-mRNA were analyzed based on the starBase^17^ (http://starbase.sysu.edu.cn/) information by using the R package ‘GDCRNATools’^18^, *p*-value < 0.05 were set as the cut-off. Then Cytoscape^19^ v3.8.2 was performed to visualize the ceRNA network of miRNA regulated the lncRNAs and mRNAs. The gene ontology (GO) analysis and the Kyoto Encyclopaedia of Genes and Genomes (KEGG) pathway enrichment analysis were performed by the R package ‘clusterProfiler^20^’, the false discovery rate (FDR) and *p*-value < 0.05 was set as the cut-off.

### Construction of prognostic prediction model

Univariate Cox hazards regression analysis was used to preliminary screen the candidate genes from the ceRNA network, *p*-value < 0.05 were set as the cut-off. Lasso regression analysis^21^ and multivariate Cox hazards regression analysis^22^ were performed to further verify the prognostic value, *p*-value < 0.05 were set as the cut-off. The multivariate Cox hazards regression coefficients were set as the β, and we calculated each case risk score with the formula: risk score = β _gene[1]_  × Expression _gene[1]_  + β_gene[2]_  × Expression _gene[2]_  + … + β_gene [n]_ × Expression _gene [n]_. Patients above the median risk score would be divided into the high-risk group, and the rest would be divided into the low-risk group. The ROC curves and K-M survival curves were utilised to evaluate the diagnostic efficacies. Finally, we constructed a nomogram to predict the prognosis of lung adenocarcinoma patients and the calibration curves were utilised to access the accuracy.

### Estimation of the immune cells in the lung adenocarcinoma

CIBERSORT algorithm^23^ was performed to estimate the component of the total immune cells in the lung adenocarcinoma and adjacent normal tissues, *p*-value < 0.05 were set as the cut-off. To visualize the composition of immune cells in the case, we used the R package ‘corplot’, ‘heatmap’ and ‘viopot’.

### Correlation analysis

We performed Cox hazards regression analysis and Lasso regression analysis to identify the survival related immune cells, *p*-value < 0.05 were set as the cut-off. Then we constructed a nomogram to predict the prognosis of the lung adenocarcinoma patients. The ROC curves and calibration curves were utilised to access the accuracy. Correlation analysis was performed for each key gene in the ceRNA network and each survival related immune cell with the Pearson method.

## Result

### Identification of the differently expressed miRNAs, lncRNAs and mRNAs

**Figure 1:**
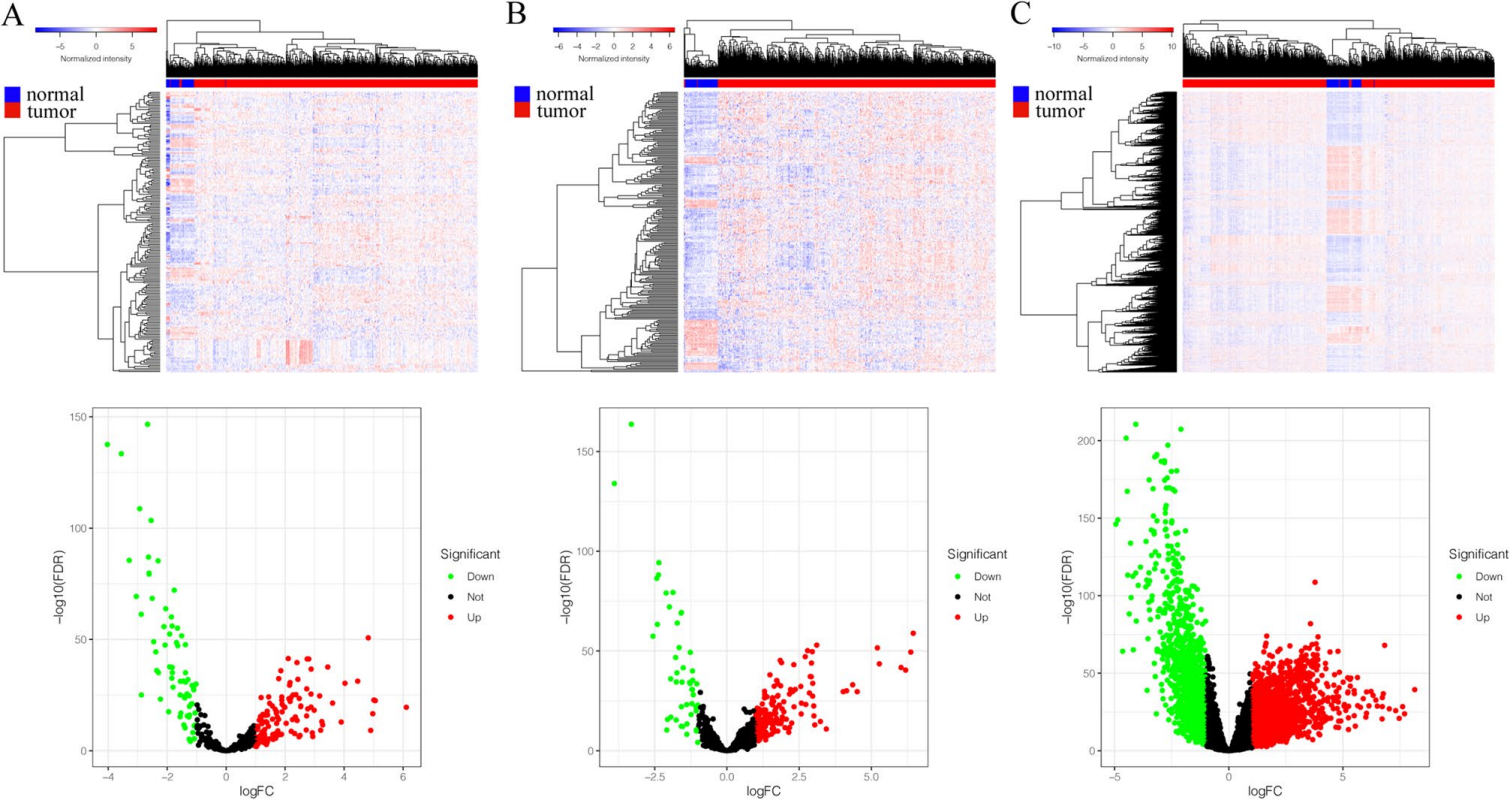
Differently expressed RNAs in TCGA lung adenocarcinoma. (**A**) The heatmap of the miRNAs expression in LUAD, and the volcano graph shows the distribution of differently expressed miRNAs. (**B**) The heatmap of the lncRNAs expression in LUAD, and the volcano graph shows the distribution of differently expressed lncRNAs. (**C**) The heatmap of the mRNAs expression in LUAD, and the volcano graph shows the distribution of differently expressed mRNAs. In the volcano graph, the X axis represents the fold changes of DEGs, and the Y axis represents the adjusted p-value, red dots present up-regulated genes and blue dots present down-regulated genes (log fold change > 1.0, *p*-value < 0.05).

All miRNA, lncRNA and mRNA profiles of lung adenocarcinoma were obtained from the TCGA. A total of 190 differently expressed miRNAs (122 up-regulated and 68 down-regulated) (Fig. 1A), 224 differently expressed lncRNAs (176 up-regulated and 48 down-regulated) (Fig. 1B) and 3024 differently expressed mRNAs (1768 up-regulated and 1256 down-regulated) (Fig. 1C) between tumor and normal tissue were identified, | logFC |> 1.0 and *p*-value < 0.05 set as the cut-off.

### Construction of the ceRNA network and functional enrichment analysis

**Figure 2:**
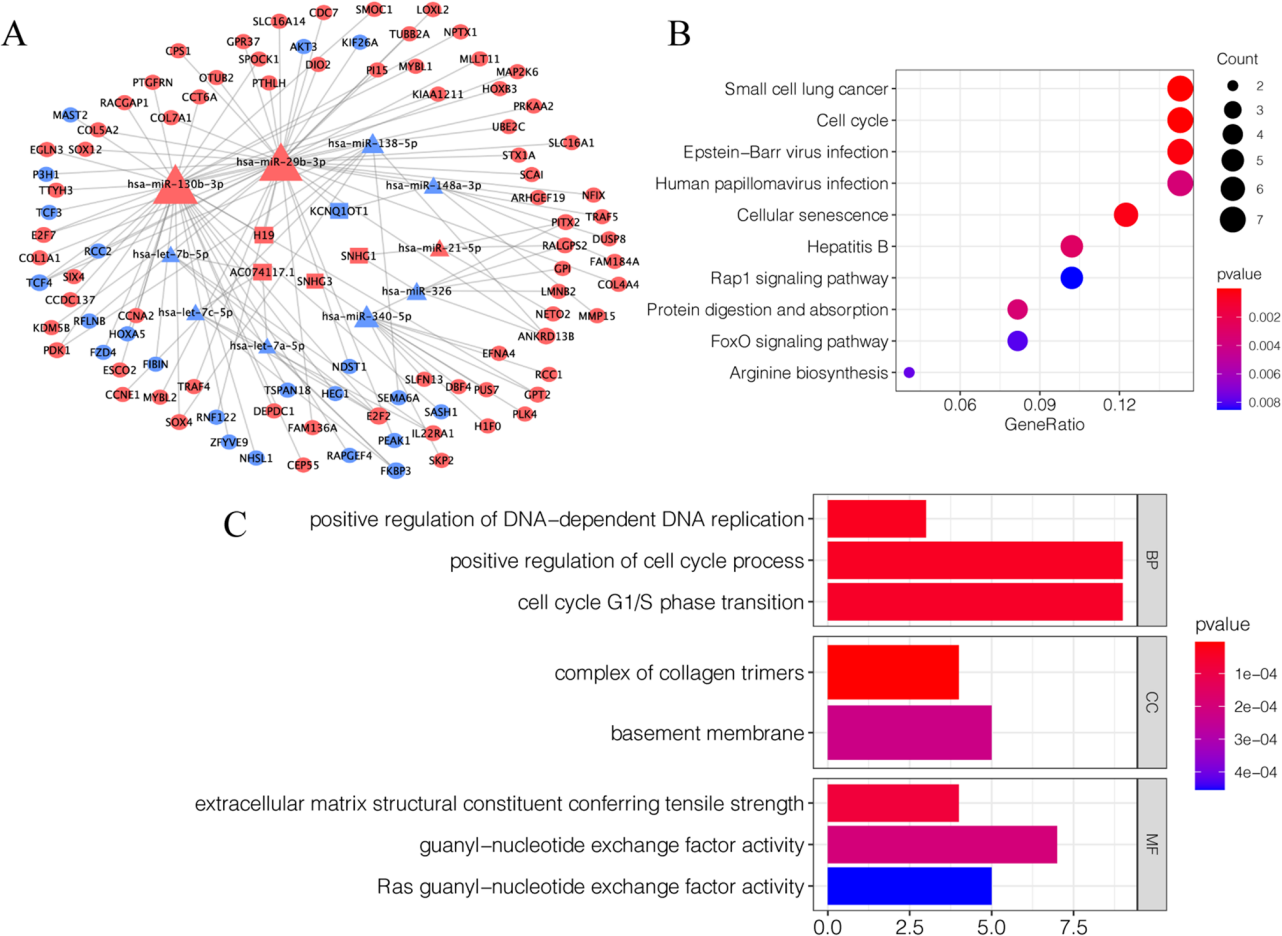
Construction of the ceRNA network and functional enrichment analysis. (**A**)The ceRNA network of the differently expressed RNAs in lung carcinoma. The triangle nodes indicate miRNAs; the rectangle nodes indicate lncRNAs; the ellipse nodes indicate mRNAs. The blue nodes indicate down-regulated genes; the red nodes indicate up-regulated genes. Node size indicates the number of the interactions. (**B**) The KEGG enrichment analysis of the ceRNA network genes. (**C**) The GO enrichment analysis of the ceRNA network genes.

Based on the information of starBase database, the lncRNA-miRNA and mRNA-miRNA interaction of the differently expressed genes was confirmed and a ceRNA network contained 5 lncRNAs, 92 mRNAs and 10 miRNAs were constructed (Fig. 2A). The KEGG pathway analysis showed that the genes in the network were primarily clustered in the small cell lung cancer, Cell cycle, Epstein-Barr virus infection and Cellular senescence (Fig. 2B). For the biological process (BP), the genes in the network were primarily enriched in the positive regulation of DNA-dependent DNA replication, positive regulation of cell cycle process and cell cycle G1/S phase transition. For the cell component (CC), the genes were primarily enriched in complex of collagen trimers and basement membrane. For the molecular function (MF), the genes were mainly enriched in extracellular matrix structural constituent conferring tensile strength, guanyl-nucleotide exchange factor activity and Ras guanyl-nucleotide exchange factor activity (Fig. 2C).

### Identification of key genes and construction of prognostic prediction model

**Figure 3:**
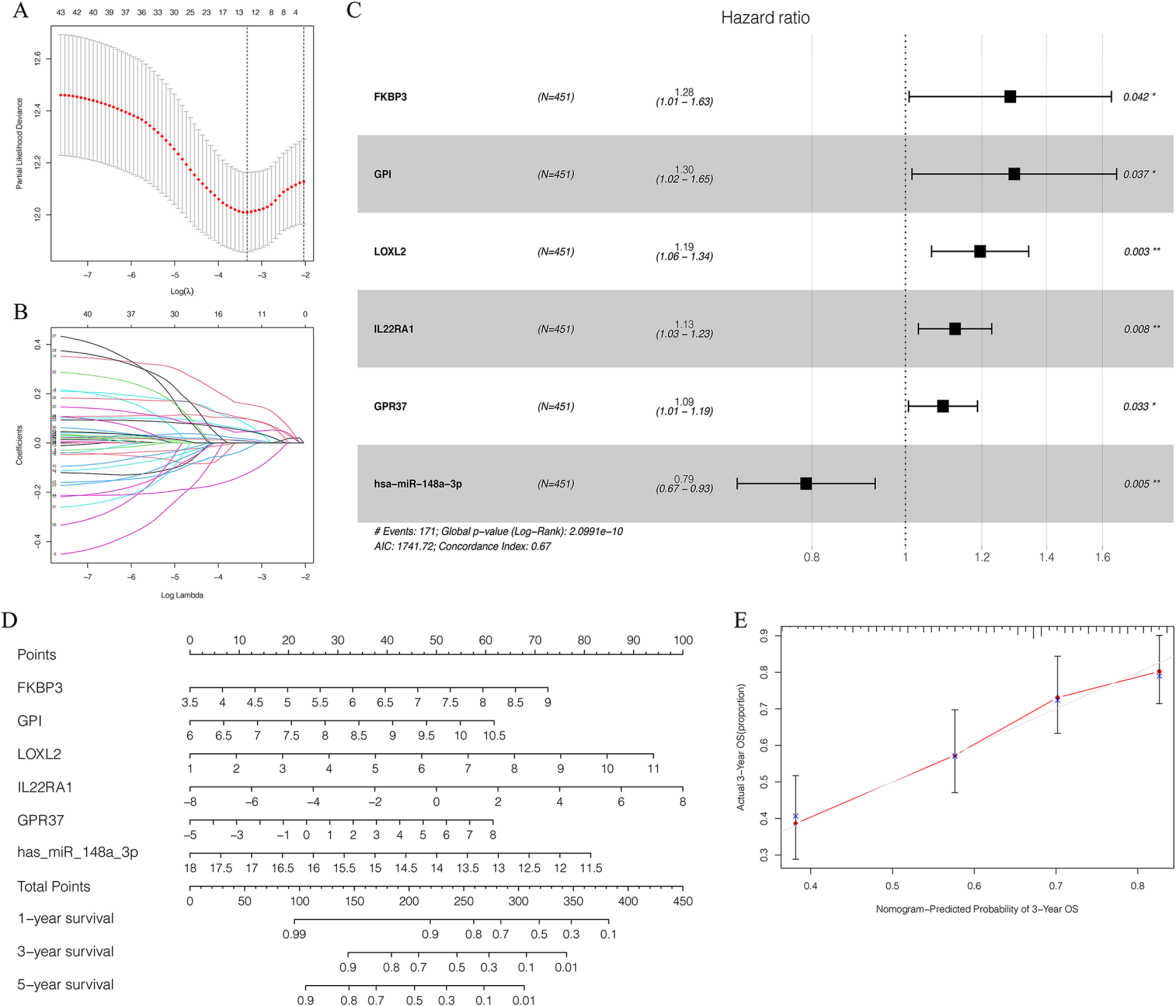
Identification of prognostic members in ceRNA network. (**A**,**B**) The Lasso regression suggested that 12 genes were essential for prognostic prediction model. (**C**) The Multivariate Cox regression analysis identified six genes (*FKBP3, GPI, LOXL2, IL22RA1, GPR37*, *has-miR-148a-3p*) were as the key gene for constructing the prognostic prediction model, *p*-value < 0.05. (**D**) The nomogram based on the key genes model, and (E) the calibration curves validated the discrimination.

**Figure 4:**
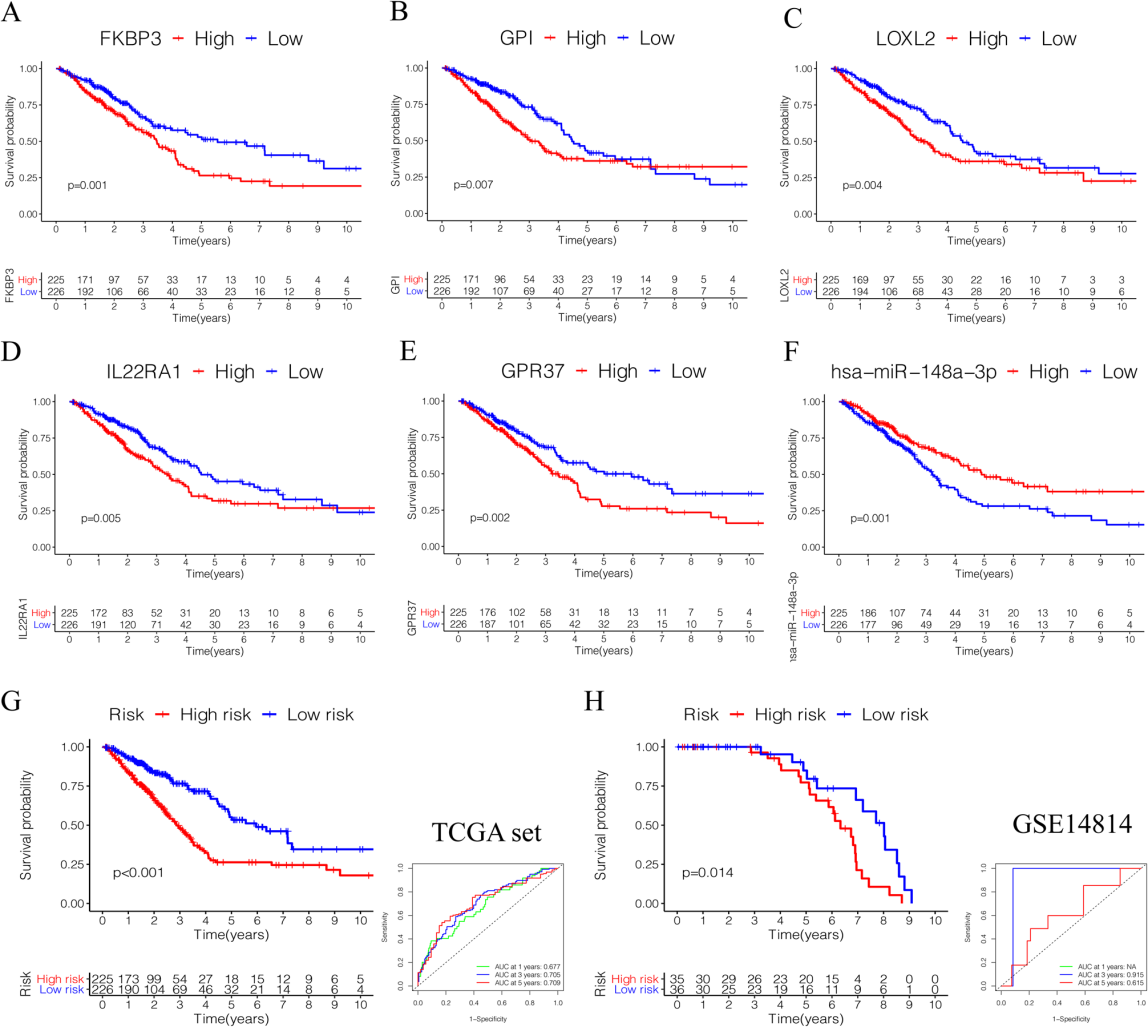
Survival analysis of prognostic genes in ceRNA network. (**A**–**F**) The Kaplan–Meier curves validated the 6 key genes (*FKBP3, GPI, LOXL2, IL22RA1, GPR37*, *has-miR-148a-3p*) were significantly associated with overall survival, *p*-value < 0.05. (**G**) The Kaplan–Meier overall survival curves for risk score groups. Red curves represent high risk group and blue curves represent low risk group, *p*-value < 0.05. (**H**) The ROC curves showed that the area under curve (AUC) at 1 year was 0.677, at 3 year was 0.705, and at 5 years was 0.709.

**Figure 5:**
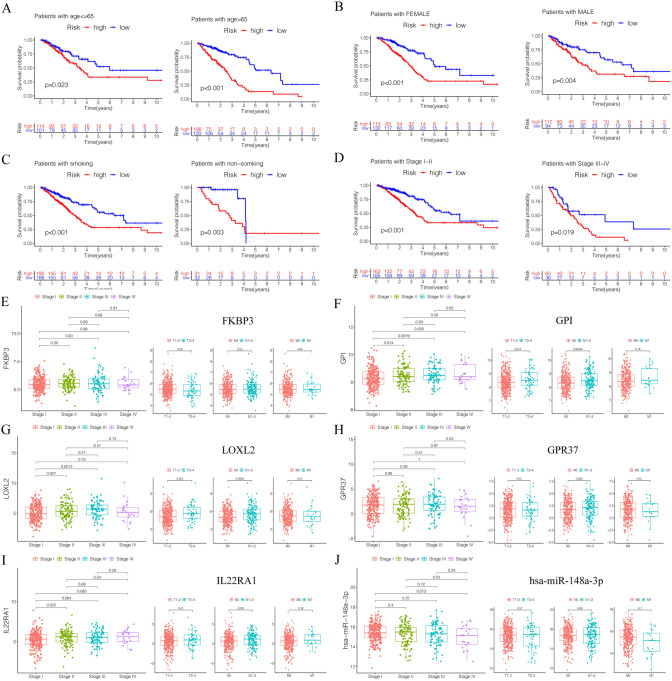
Subgroup analysis classified by patients' age (**A**), gender (**B**), smoking status (**C**) and clinic stage (**D**) were conducted. (**E**–**J**) The expression of the 6 key genes (*FKBP3, GPI, LOXL2, IL22RA1, GPR37*, *has-miR-148a-3p*) in lung adenocarcinoma patients with different tumor stages, T stages, N stages and M stages.

All clinicopathological characteristics of patients in TCGA lung adenocarcinoma were summarized in Table 1. Univariate Cox hazards regression analysis detected 45 candidate genes from the ceRNA network (Supplementary Table) and the lasso regression analysis filtered 12 genes were reliable for prediction model, *p*-value < 0.05 set as the cut-off (Fig. 3A,B). Then multivariate Cox hazards regression analysis identified 6 genes (*FKBP3, GPI, LOXL2, IL22RA1, GPR37, has-miR-148a-3p*) were as the key members for constructing the prognostic prediction model in the in the network, *p*-value < 0.05 set as the cut-off (Fig. 3C). Based on the model, a nomogram was constructed and a good discrimination of the model was validated by the calibration curves (Fig. 3D,E). Kaplan–Meier curves validated these 6 key genes (Fig. 4A–F) and the model were significantly associated with overall survival, *p*-value < 0.05 set as the cut-off (Fig. 4G). The ROC curves showed that the area under curve (AUC) at 1 year was 0.677, at 3 year was 0.705, and at 5 years was 0.709 (Fig. 4H). We used GSE14814 lung adenocarcinoma data sets (n = 71) to evaluate the prognostic value of the model. The Kaplan–Meier curves validated the model were significantly associated with overall survival, *p*-value < 0.05, and the AUC at 3 year was 0.915, at 5 year was 0.615(Fig. 4G). Subgroup analysis classified by patients' age, gender, smoking status or clinic stage were subsequently conducted, the results revealed that the high- risk group in this model were significantly associated with worse prognosis among all the subgroups (Fig. 5A–D). The expression of the 6 key genes (*FKBP3, GPI, LOXL2, IL22RA1, GPR37, has-miR-148a-3p*) in lung adenocarcinoma patients with different tumor stages, T stages, N stages and M stages (Fig. 5E–J).

**Table 1:** Clinicopathological characteristics of patients in TCGA lung carcinoma.

**Age**	Cases (%)
≤ 65	215 (44.2%)
> 65	226 (46.6%)
Unknown	45 (9.2%)
**Gender**	
Male	222 (47.44%)
Female	264 (56.41%)
**Smoking history**	
Smoked	284 (60.68%)
Non- smoked	190 (40.60%)
Unknow	12 (2.56%)
**Clinical stage**	
I	262 (55.98%)
II	112 (23.93%)
III	79 (16.88%)
IV	25 (5.34%)
Unknow	8 (1.71%)
**T _stage**	
T1	163 (34.83%)
T2	260 (55.56%)
T3	41 (8.76%)
T4	19 (4.06%)
Tx/Unknow	3 (0.64%)
**N_stage**	
N0	312 (66.67%)
N1	90 (19.23%)
N2	70 (14.96%)
N3	2 (0.43%)
Nx/Unknow	12 (2.56%)
**M_stage**	
M0	333 (71.15%)
M1	24 (5.13%)
Mx/Unknow	129 (27.56%)

### Comprehensive analysis of immune cells and prognostic value in lung adenocarcinoma

**Figure 6:**
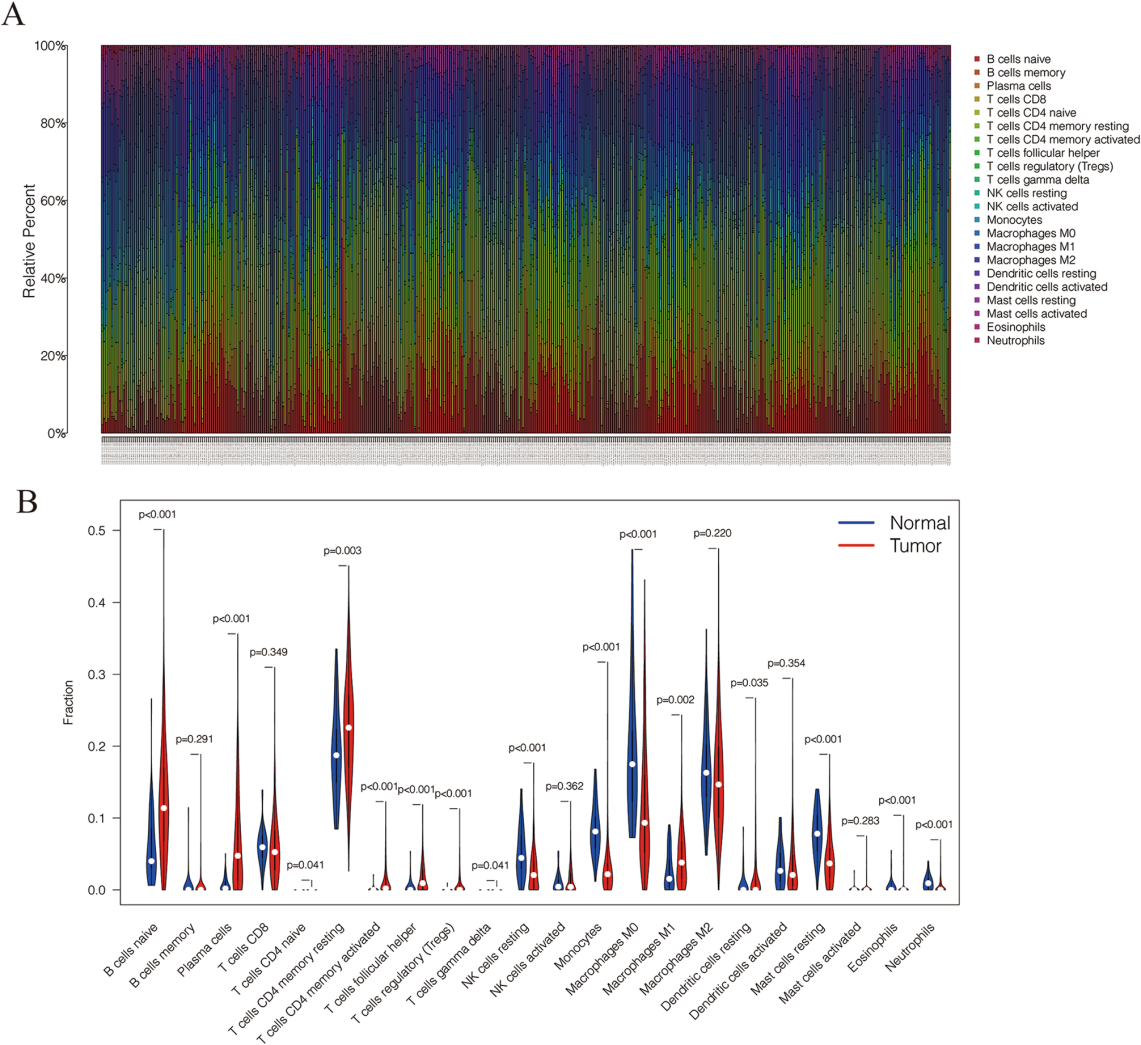
The composition of immune cells in lung adenocarcinoma. (**A**) The component of immune cells in the TCGA-LUAD cases. (**B**) The violin plot shows the levels of immune cells in the tumor group (red) and the normal tissue group (blue).

**Figure 7:**
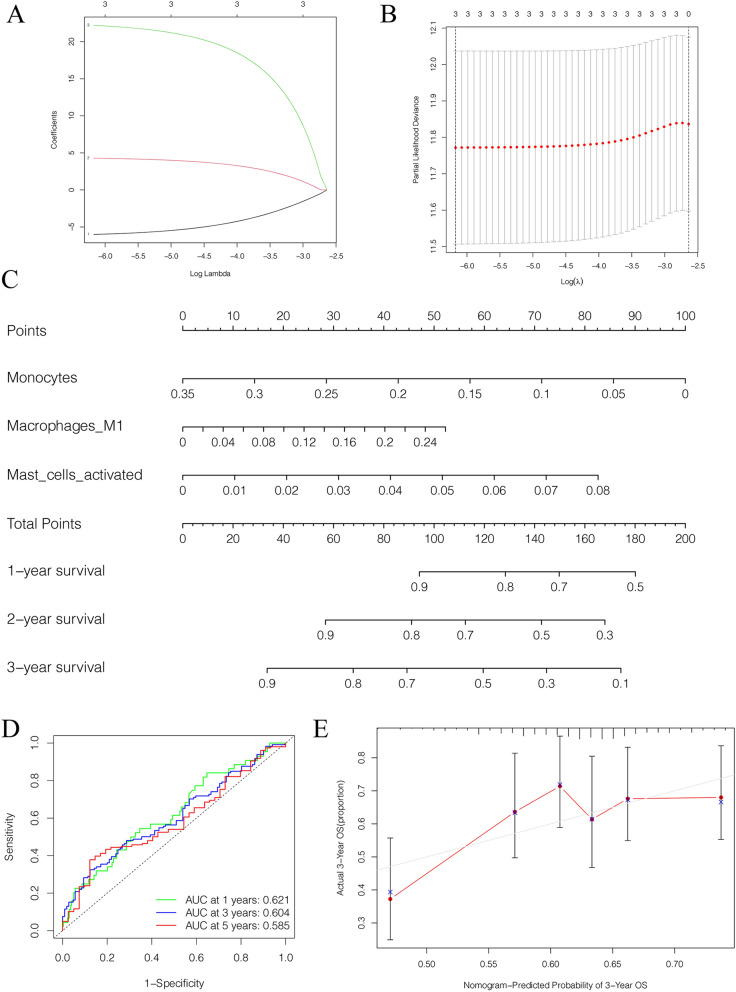
Identification of prognostic value of immune cells in lung adenocarcinoma. (**A**,**B**) The Lasso regression suggested that 3 immune cells were essential for prognostic prediction model. (**C**) The nomogram based on the immune cells model. (**D**) The ROC curves showed that the area under curve at 1 year was 0.621, at 3 year was 0.604, and at 5 years was 0.585. (**E**) The calibration curves validated the discrimination.

The CIBERSORT algorithm estimated the component of immune cells in lung adenocarcinoma (Fig. 6A,B). Cox hazards regression analysis detected 3 immune cells (Monocytes, Macrophages M1, activated mast cells) were associated with the prognosis in lung adenocarcinoma (Table 2), and the lasso regression analysis validated the 3 immune cells were reliable for prediction as an immune cell model, *p*-value < 0.05 set as the cut-off (Fig. 7A,B). Based on the result, a nomogram was constructed (Fig. 7C). The ROC curves showed that the area under curve (AUC) at 1 year was 0.621, at 3 year was 0.604, and at 5 years was 0.585 (Fig. 7D). The discrimination of the 3 immune cells were validated by the calibration curves (Fig. 7E).

**Table 2:** Univariate cox hazards regression analysis of immune cells.

Immune cell	HR	95%CI	*p*-value
Monocytes	6.31E−04	(1.02E−06—3.88E−01)	0.0246
Macrophages M1	1.09E + 02	(1.67E + 00—7.09E + 03)	0.0276
Activated mast cells	4.03E + 09	(1.02E + 02—1.58E + 17)	0.0132
B cells naive	4.71E−01	(5.56E−02—3.99E + 00)	0.4902
B cells memory	1.13E + 02	(1.66E−01—7.70E + 04)	0.1555
Plasma cells	4.15E−01	(3.27E−02—5.27E + 00)	0.4975
T cells CD8	1.36E−01	(2.51E−03—7.41E + 00)	0.3284
Memory resting T cells CD4	3.41E−01	(3.11E−02—3.74E + 00)	0.3787
Memory activated T cells CD4	5.61E−01	(4.51E−05—6.96E + 03)	0.9042
T cells follicular helper	6.33E + 00	(4.08E−03—9.81E + 03)	0.6225
T cells regulatory (Tregs)	1.35E + 03	(1.31E−01—1.40E + 07)	0.1263
Resting NK cells	3.11E + 00	(5.66E−03—1.71E + 03)	0.7246
Activated NK cells	2.46E + 02	(3.26E−02—1.86E + 06)	0.2268
Macrophages M0	1.92E + 00	(3.23E−01—1.14E + 01)	0.4728
Macrophages M2	1.32E + 00	(1.71E−01—1.02E + 01)	0.7891
Resting dendritic cells	8.08E−02	(4.03E−04—1.62E + 01)	0.3523
Activated dendritic cells	1.82E + 01	(2.33E−01—1.42E + 03)	0.1920
Resting mast cells	6.40E−01	(2.57E−03—1.60E + 02)	0.8741
Eosinophils	1.37E + 13	(1.53E−07—1.23E + 33)	0.1968
Neutrophils	6.16E + 01	(6.90E−07—5.50E + 09)	0.6591

*HR* hazards regression, *CI* confidence interval.

### Correlation analysis between the key genes of ceRNA network and the prognostic immune cells

**Figure 8:**
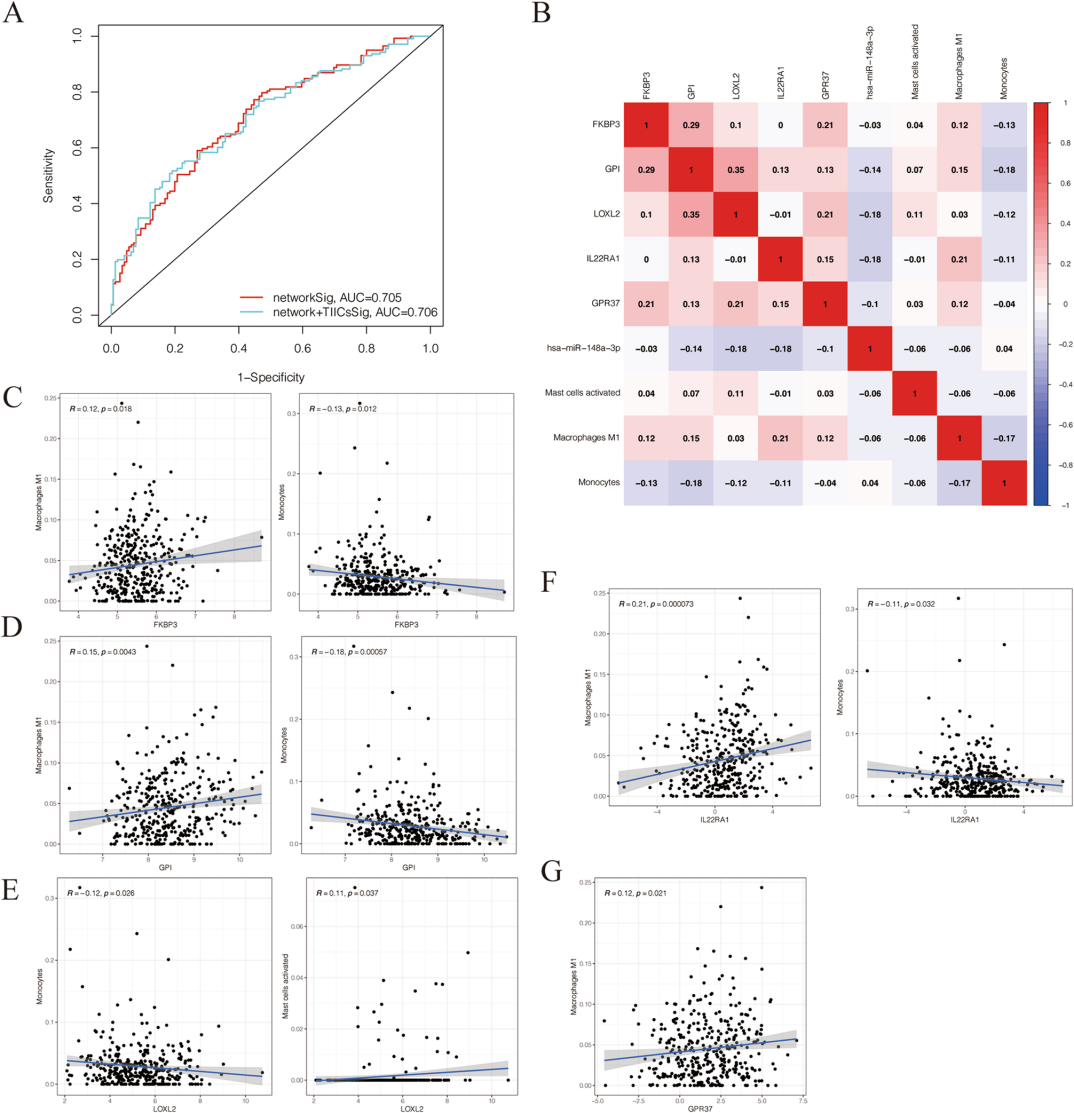
Correlation analysis between the key genes of ceRNA network and the prognostic immune cells. (**A**) The ROC curves of the ceRNA network signatures and ceRNA network combined TIICs signatures. (**B**) The co-expression heatmap of all key genes and the prognostic immune cells. (**C**) The *FKBP3* was associated with Monocytes and Macrophages M1. (**D**) The *GPI* was associated with Monocytes and Macrophages M1. (**E**) The *LOXL2* was negatively associated with Monocytes and Activated mast cells. (**F**) The *IL22RA1* was related with Monocytes and Macrophages M1. (**G**) The *GPR37* was associated with Macrophages M1. R, correlation coefficients. *p*-value < 0.05.

The ROC analysis at 3 year performed for comparison the effect of the ceRNA network and the combination of TIICs. The results showed that the AUC for network and network + TIICs is 0.705 and 0.706, respectively (Fig. 8A). Pearson correlation analysis indicated that the *FKBP3* was negatively associated with Monocytes (R = − 0.13, *p* < 0.05) and positively associated with Macrophages M1 (R = 0.12, *p* < 0.05) (Fig. 8C). The *GPI* was negatively associated with Monocytes (R = − 0.18, *p* < 0.05) and positively associated with Macrophages M1 (R = 0.15, *p* < 0.05) (Fig. 8D). The *LOXL2* was negatively associated with Monocytes (R = − 0.12, *p* < 0.05) and positively associated with activated mast cells (R = 0.11, *p* < 0.05) (Fig. 8E). The *IL22RA1* was negatively associated with Monocytes (R = − 0.11, *p* < 0.05) and positively associated with Macrophages M1 (R = 0.21, *p* < 0.05) (Fig. 8F). Moreover, the *GPR37* was positively associated with Macrophages M1 (R = 0.12, *p* < 0.05) (Fig. 8G). The correlation of all key genes and the prognostic immune cells were summarized in the heatmap (Fig. 8B).

## Discussion

Lung cancer is a heterogeneous disease which is still a big challenge to treatment. Even a part of patients with early-stage tumors who received surgery ultimately develop recurrence^24^. In the process of tumor occurrence and progression, molecular components play an important role and are generally considered as potential prognostic factors^25^. Tumor infiltrating immune cells have been proved to be very important in the occurrence and development of tumors, and have shown the prognostic value of a variety of malignant tumors^26–28^.

Generally, the ceRNA regulatory networks include the lncRNA, miRNA, circular RNA and mRNA^29^. Previous studies have shown that the non-coding RNAs play a vital role in cancer occurrence and prognosis, and it has also been generally recognized that the interactions between lncRNA, miRNA and mRNA could regulate the expression levels of mRNAs and the affect the core protein signals, inducing the changes in physiological functions of cells^14^. In this study, we detected the differently expressed miRNAs, lncRNAs and mRNAs in the TCGA lung adenocarcinoma for the first and constructed a ceRNA network from these differently expressed genes.

To identify the key biomarker for prognosis, we performed Cox hazards regression analysis, Lasso regression analysis and survival analysis. Then, *FKBP3, GPI, LOXL2, IL22RA1, GPR37* and *has-miR-148a-3p* were identified as the prognostic biomarkers that were significantly associated with overall survival of LUAD patients. In previous studies, these 6 key genes have been confirmed to play an important regulatory role in the progression of tumors. Zhu et al. have provided evidence that *FKBP3* plays an essential role in proliferation and cell cycle progression of NSCLC and might be valued as a prognostic marker in NSCLC^30^. *IL22RA1* is a receptor of interleukin 22, has been reported working as a signal transducer and activator of STAT3 signaling in the malignant transformation, it plays an important role in promoting tumor growth and metastasis and inhibiting cell apoptosis^31–33^. Besides, many studies have shown that the abnormal expression of *LOXL2* in a variety of cancers is related to epithelial-mesenchymal transition, metastasis, poor prognosis, resistance to radiotherapy and chemotherapy, and tumor progression^34^. Consistent with our result, a previous study demonstrated that *LOXL2* levels are positively associated with poor prognosis in NSCLC patients. In addition, *GPR37* also has been shown as a poor prognostic factor in LUAD patients with TP53 mutation combined EGFR mutation^35^.

Next, we estimated tumor-infiltrating immune cells through the CIBERSORT algorithm, and used Cox hazards regression analysis and lasso regression analysis to identify prognostic immune cells. Our results show that the Monocytes, Macrophages M1 and Activated mast cells identified were significantly related with survival. Previous studies proved that Monocytes and macrophages play an important role in the immune system, Monocyte activation and then macrophage differentiation is an important step for immune responses^36^. In the early stages of tumor progression, Monocyte’s recruitment can be found in many types of cancer, and Monocytes induce direct killing of malignant cells through cytokine-mediated cell death and phagocytosis^37,38^. Macrophages M1 is one major type cell subtype of tumor-associated macrophages (TAMs) which are an important part of the tumor microenvironment, and the infiltration of TAMs is closely related to the poor survival of patients with solid tumors^39,40^. Cytokines secreted by TAMs induce anti-apoptotic programs in cancer cells, such as *IL-6* derived from TAMs that mediate the resistance of solid tumors to many chemotherapies via activating TGF-β pathway^41–43^. Although Mast cells participate in the maintenance of healthy lungs through innate immunity and adaptive immunity^44^, there is evidence that mast cell activation leads to the proliferation of non-small cell lung cancer cells^45^.

According to previous study, Kim et. al. proved that the *IL-22* could promote the infiltrating immune cells, including macrophages, and then regulate the inflammatory processes^14^. Our correlation analysis revealed that the correlation between *IL22RA1* with macrophage was significantly obvious suggesting that the *IL22RA1* might be an immune-related gene in lung carcinoma. In addition, the correlation analysis revealed that the *GPR37* were also associated with immune cells. Therefore, our results support that the crosstalk between the ceRNA network and tumor-infiltrating immune cells might play a vital role in the formation and progression of lung adenocarcinoma.

In conclusion, through comprehensive analysis, we constructed a ceRNA network and identified *FKBP3, GPI, LOXL2, IL22RA1, GPR37* and *has-miR-148a-3p* as the prognostic members of the network, and then identified Monocytes, Macrophages M1 and activated Mast cells as the prognostic tumor-infiltrating immune cells, thus constructing two nomograms that can be used as indicators for evaluating the survival of lung adenocarcinoma patients. Moreover, our results also suggest the crosstalk between the ceRNA network and tumor-infiltrating immune cells to explore the molecular mechanisms involved in lung adenocarcinoma.

## Supplementary Information

Supplementary Information.

## Data Availability

All the data used in this study were downloaded from The Cancer Genome Atlas portal (TCGA, https://portal.gdc.cancer.gov/).

## References

[1] Siegel RL, Miller KD, Jemal A (2020). Cancer statistics, 2020. CA Cancer J. Clin..

[2] Herbst RS, Morgensztern D, Boshoff C (2018). The biology and management of non-small cell lung cancer. Nature.

[3] Tanoue LT, Tanner NT, Gould MK, Silvestri GA (2015). Lung cancer screening. Am. J. Respir. Crit. Care Med..

[4] Duma N, Santana-Davila R, Molina JR (2019). Non-small cell lung cancer: Epidemiology, screening, diagnosis, and treatment. Mayo Clin. Proc..

[5] Hirsch FR (2017). Lung cancer: Current therapies and new targeted treatments. Lancet.

[6] Guo LL (2015). Competing endogenous RNA networks and gastric cancer. World J. Gastroenterol..

[7] Tay Y, Rinn J, Pandolfi PP (2014). The multilayered complexity of ceRNA crosstalk and competition. Nature.

[8] Karreth FA, Pandolfi PP (2013). ceRNA cross-talk in cancer: when ce-bling rivalries go awry. Cancer Discov..

[9] Weng W (2020). Identification of a competing endogenous RNA network associated with prognosis of pancreatic adenocarcinoma. Cancer Cell Int..

[10] Emilsson V (2008). Genetics of gene expression and its effect on disease. Nature.

[11] Singh R, Mishra MK, Aggarwal H (2017). Inflammation, immunity, and cancer. Mediators Inflamm..

[12] Grivennikov SI, Greten FR, Karin M (2010). Immunity, inflammation, and cancer. Cell.

[13] Kardoust Parizi M, Shariat SF, Margulis V, Mori K, Lotan Y (2020). Value of tumour-infiltrating immune cells in predicting response to intravesical BCG in patients with non-muscle-invasive bladder cancer: A systematic review and meta-analysis. BJU Int..

[14] Raziq K (2020). Competitive endogenous network of lncRNA, miRNA, and mRNA in the chemoresistance of gastrointestinal tract adenocarcinomas. Biomed. Pharmacother..

[15] Zhu CQ (2010). Prognostic and predictive gene signature for adjuvant chemotherapy in resected non-small-cell lung cancer. J. Clin. Oncol..

[16] Anders S, Huber W (2010). Differential expression analysis for sequence count data. Genome Biol..

[17] Li JH, Liu S, Zhou H, Qu LH, Yang JH (2014). starBase v2.0: decoding miRNA-ceRNA, miRNA-ncRNA and protein-RNA interaction networks from large-scale CLIP-Seq data. Nucleic Acids Res..

[18] Li R (2018). GDCRNATools: An R/Bioconductor package for integrative analysis of lncRNA, miRNA and mRNA data in GDC. Bioinformatics.

[19] Shannon P (2003). Cytoscape: A software environment for integrated models of biomolecular interaction networks. Genome Res..

[20] Yu G, Wang LG, Han Y, He QY (2012). clusterProfiler: An R package for comparing biological themes among gene clusters. OMICS.

[21] Colombani C (2013). Application of Bayesian least absolute shrinkage and selection operator (LASSO) and BayesCpi methods for genomic selection in French Holstein and Montbeliarde breeds. J. Dairy Sci..

[22] Cox DR (1972). Regression models and life-tables. J. Roy. Stat. Soc.: Ser. B (Methodol.).

[23] Newman AM (2015). Robust enumeration of cell subsets from tissue expression profiles. Nat Methods.

[24] Sata Y (2019). Keys to successful induction chemoradiotherapy followed by surgery for stage III/N2 non-small cell lung cancer. Surg. Today.

[25] Zanconato F, Cordenonsi M, Piccolo S (2019). YAP and TAZ: A signalling hub of the tumour microenvironment. Nat. Rev. Cancer.

[26] Tsou P, Katayama H, Ostrin EJ, Hanash SM (2016). The emerging role of B cells in tumor immunity. Cancer Res..

[27] Takeuchi Y, Nishikawa H (2016). Roles of regulatory T cells in cancer immunity. Int. Immunol..

[28] Jochems C, Schlom J (2011). Tumor-infiltrating immune cells and prognosis: The potential link between conventional cancer therapy and immunity. Exp. Biol. Med. (Maywood).

[29] Poliseno L (2010). A coding-independent function of gene and pseudogene mRNAs regulates tumour biology. Nature.

[30] Zhu W (2017). FKBP3 promotes proliferation of non-small cell lung cancer cells through regulating Sp1/HDAC2/p27. Theranostics.

[31] Cui X (2019). IL22 furthers malignant transformation of rat mesenchymal stem cells, possibly in association with IL22RA1/STAT3 signaling. Oncol. Rep..

[32] He W (2018). IL22RA1/STAT3 signaling promotes stemness and tumorigenicity in pancreatic cancer. Cancer Res..

[33] Trevejo-Nunez G, Elsegeiny W, Conboy P, Chen K, Kolls JK (2016). Critical role of IL-22/IL22-RA1 signaling in pneumococcal pneumonia. J. Immunol..

[34] Wen B, Xu LY, Li EM (2020). LOXL2 in cancer: Regulation, downstream effectors and novel roles. Biochim. Biophys. Acta Rev. Cancer.

[35] Wang F (2020). Prognostic value of TP53 co-mutation status combined with EGFR mutation in patients with lung adenocarcinoma. J. Cancer Res. Clin. Oncol..

[36] Ivanova EA, Orekhov AN (2016). Monocyte activation in immunopathology: Cellular test for development of diagnostics and therapy. J. Immunol. Res..

[37] Olingy CE, Dinh HQ, Hedrick CC (2019). Monocyte heterogeneity and functions in cancer. J. Leukoc. Biol..

[38] Woods JA, Davis JM (1994). Exercise, monocyte/macrophage function, and cancer. Med. Sci. Sports Exerc..

[39] Singhal S (2019). Human tumor-associated monocytes/macrophages and their regulation of T cell responses in early-stage lung cancer. Sci. Transl. Med..

[40] Yang L, Zhang Y (2017). Tumor-associated macrophages: From basic research to clinical application. J. Hematol. Oncol..

[41] Kong L (2016). Deletion of interleukin-6 in monocytes/macrophages suppresses the initiation of hepatocellular carcinoma in mice. J. Exp. Clin. Cancer Res..

[42] Xu X, Ye J, Huang C, Yan Y, Li J (2019). M2 macrophage-derived IL6 mediates resistance of breast cancer cells to hedgehog inhibition. Toxicol. Appl. Pharmacol..

[43] Yin Y (2017). The immune-microenvironment confers chemoresistance of colorectal cancer through macrophage-derived IL6. Clin. Cancer Res..

[44] Virk H, Arthur G, Bradding P (2016). Mast cells and their activation in lung disease. Transl. Res..

[45] Stoyanov E, Uddin M, Mankuta D, Dubinett SM, Levi-Schaffer F (2012). Mast cells and histamine enhance the proliferation of non-small cell lung cancer cells. Lung Cancer.

